# Interactions between APP secretases and inflammatory mediators

**DOI:** 10.1186/1742-2094-5-25

**Published:** 2008-06-18

**Authors:** Magdalena Sastre, Jochen Walter, Steve M Gentleman

**Affiliations:** 1 Division of Neuroscience and Mental Health, Imperial College London, The Hammersmith Hospital, Du cane Road, London W12 0NN, UK; 2 Department of Neurology, University Bonn, Bonn, Germany

## Abstract

There is now a large body of evidence linking inflammation to Alzheimer's disease (AD). This association manifests itself neuropathologically in the presence of activated microglia and astrocytes around neuritic plaques and increased levels of inflammatory mediators in the brains of AD patients. It is considered that amyloid-β peptide (Aβ), which is derived from the processing of the longer amyloid precursor protein (APP), could be the most important stimulator of this response, and therefore determining the role of the different secretases involved in its generation is essential for a better understanding of the regulation of inflammation in AD. The finding that certain non-steroidal anti-inflammatory drugs (NSAIDs) can affect the processing of APP by inhibiting β- and γ-secretases, together with recent revelations that these enzymes may be regulated by inflammation, suggest that they could be an interesting target for anti-inflammatory drugs. In this review we will discuss some of these issues and the role of the secretases in inflammation, independent of their effect on Aβ formation.

## Introduction

Alzheimer's disease (AD) is a devastating neurological disease affecting more than 26 million people around the world, and there are indications that this number will quadruple by 2050. Age is the most significant risk factor and it is estimated that 50% of people over 85 have either AD or Mild Cognitive Impairment (MCI). Brains of individuals with AD manifest massive neuronal and synaptic loss in certain areas which result in the memory impairment and disorientation associated with this disease. The neuroanatomical study of a typical AD brain reveals the presence of two characteristic lesions: extracellular amyloid (or senile plaques) and intracellular neurofibrillary tangles composed primarily of hyperphosphorylated tau protein [1]. Amyloid plaques contain small amyloid-β peptides (Aβ), which are toxic products from the catalytic cleavage of a larger amyloid precursor protein (APP) [1,2].

In 1907 *Alois Alzheimer *described unique structures in the cerebral cortex of a 55 year old woman with progressive dementia that are now referred to as senile plaques [3]. Histological methods used to demonstrate plaques including thioflavine S, various silver stains, and immunocytochemical stains reveal various biochemical components of plaques. Aβ has been reported to have heterogeneous carboxyl termini, and Aβ1–40 and Aβ1–42 appear to be the major species in the parenchymal deposits. It is this C-terminal variation that has been most often associated with pathogenicity, with Aβ1–42 found to be the most toxic form [1,2]. Aβ is found in normal cerebrospinal fluid and in conditioned media from various tissue culture cell lines [4-6], suggesting that it is produced and secreted constitutively.

Aβ deposition is considered a major pathogenic step in the development of AD. Evidence supporting the amyloid hypothesis has been extensively reviewed [7]. On the other hand, there is also the view that Aβ plaque formation is an epiphenomenon and its pathophysiological functions still remain unclear. In this regard, it has been shown that in people with AD the density of Aβ plaques correlates poorly with the severity of dementia [8]. There are, however, indications that dendritic and synaptic injury occur early in the course of the disease and that protofibrils and oligomers of Aβ40 and Aβ42 could cause neuronal dysfunction [9]. In addition, a recent study carried out using multiphoton laser confocal microscopy showed that in the early stages of the disease, microplaques can damage neighbouring axons and dendrites within days [10]. Furthermore, intraneuronal Aβ has also been implicated in the onset of cognitive dysfunction [11].

**Figure 1:**
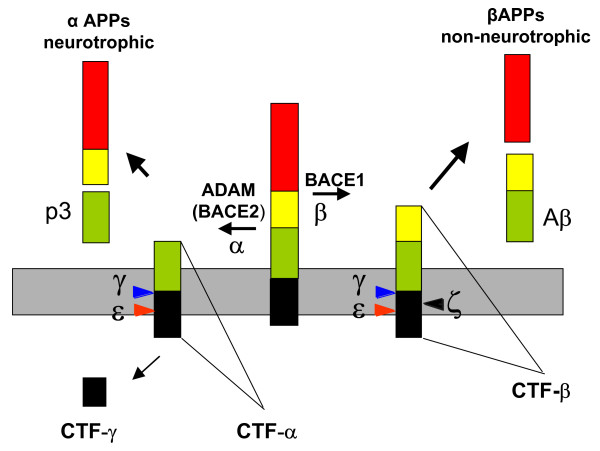
Proteolytic processing of APP. Proteolysis of APP by α-secretase or β-secretase leads to the secretion of αAPPs or βAPPs. Both secretases generate C-terminal fragments (CTF) of 10 kDa and 12 kDa respectively, which are inserted in the membrane (grey). These fragments can be cut by γ-secretase to release the peptides P3 and Aβ. Two further cleavage sites, termed ε and ζ, have been identified in the CTF.

APP is a type I integral membrane protein [12] that resembles a signal-transduction receptor. It is synthesized in the ER, post-transcriptionally modified in the Golgi (including N- and O-linked glycosylation, sulfation and phosphorylation), and transported to the cell surface via the secretory pathway. APP is also endocytosed from the cell surface and processed in the endosomal-lysosomal pathway [13,14], although autophagic vacuoles may also be a site for Aβ production [15]. Alternative processing pathways of APP have been described (Figure 1).

Proteolysis of APP by α-secretase or β-secretase leads to the secretion of soluble α-APPs or β-APPs. This generates C-terminal fragments of 10 kDa and 12 kDa respectively, which are inserted in the membrane. These fragments can be cut by γ-secretase to release the peptides P3 and Aβ [16] and a cytoplasmic fragment identified as AICD (APP intracellular domain) [17]. Intriguingly, AICD starts at position 49/50 and would not correspond to the end of Aβ variants Aβ40 and Aβ42. Therefore this cleavage site, termed ε-cleavage, is topologically highly similar to the S3 cleavage of Notch [17,18]. Recently, a new cleavage site was described for γ-secretase. The ξ-cleavage occurs between ε- and γ-cleavage sites and generates longer Aβ forms within cells and in the brain, including Aβ43, Aβ45, Aβ46, and Aβ48 [19,20].

## Inflammation and Alzheimer's disease

There is strong evidence that Aβ toxicity could be mediated through the induction of inflammatory events in the brain. Over the past decade it has been speculated that the inflammatory response associated with the presence of neuritic plaques could be involved in neuronal damage and contribute to the progression of the disease [21,22]. A common feature in the brain of AD patients is the presence of astrocytes and microglia surrounding the senile amyloid plaques, already described by Alois Alzheimer in 1907.

The first evidence for an excessive inflammatory process in AD came from a study carried out in AD and Down syndrome brains that showed increased levels of S100 and IL-1 [23]. Since then, many of the cytokines and chemokines that have been studied in AD, including IL-1β, IL-6, TNF-α, IL-8, TGF-β and macrophage inflammatory protein-1α (MIP-1α) have been found to have altered expression compared with control individuals [22]. Animal models of Alzheimer's disease, such as the APP transgenic line Tg2567 carrying the Swedish mutation, also show enhanced levels for TNF-α, IL-1β, IL-1α, chemoattractant protein-1, COX-2 and complement component 1q [24,25]. In addition, an increased risk of AD has been associated with several polymorphisms of proinflammatory genes, including IL-1 [26], IL-6 [27], TNF-α [28], and α1-antichymotrypsin [29].

Clinical investigation and studies in AD animal models have reinforced the suggestion that inflammatory changes in the AD brain are an early and prominent feature. In support of this hypothesis, imaging studies have detected microglial activation in patients at very early clinical stages of the disease [30]. Moreover, a recent study investigating predictors in plasma from MCI patients and those with pre-symptomatic AD that converted to AD identified factors associated with inflammation, such as TNFα, IL-3 IL-1α and IL-11 [31]. In animal models of AD, focal glial activation already takes place before amyloid plaque formation in APPV717 transgenic mice at 3 months of age [32]. These animals showed decreased LTP already at this age, which could be caused by impaired neuronal function derived from the increased secretion of pro-inflammatory cytokines [33]. Prominent microglial activation also preceded tangle formation in 3 month old P301S Tg mice [34]. Interestingly, neuroinflammation has been proposed to be the link between Aβ deposition and the formation of neurofibrillary tangles. Products of inflammation, such as pro-inflammatory cytokines might change the substrate specificity of kinases/phosphatases leading to tau phosphorylation at pathological sites [35].

Further evidence for a link between inflammation and AD comes from observations in head-injured patients. A number of large epidemiological studies (e.g. the MIRAGE study) [36] have identified an increased risk of dementia in patients who have suffered a serious head injury during their life. At the more acute pathological level, in a cohort of patients who died within weeks of their head injury, approximately a third showed signs of Aβ deposition in their brains [37]. While there was a wide age range in this cohort and some of these Aβ deposits were almost certain to predate the trauma, the deposits seen in the younger cases are likely to have been generated de novo, as a result of the trauma. We have hypothesised that it is the inflammatory response generated as a result of the trauma which triggers the AD-type degenerative changes [38]. Traumatic brain injury in rats has been shown to increase BACE1 activity in the hippocampus with a concomitant increase in APP cleavage products [39]. Work in a porcine model of head injury has shown accumulation of APP and BACE in injured axons and it is suggested that the abnormal accumulation of these proteins may favour Aβ production [40]. At the molecular level acute hypoxia has been shown to increase the production of APP and subsequently Aβ. This action is thought to be mediated by the binding of HIF-1 to the promoter region of BACE1 mRNA resulting in increased levels of BACE1 protein in the tissue [41]. More recently it has been shown that brain injury in mice increases the expression of presenilin 1 and nicastrin, both components of the γ-secretase complex, in activated microglia and astrocytes [42].

Conversely, it has to be noted that inflammation is not only contributing to the disease progression, but could have beneficial effects. This may depend on the inflammatory elements activated, the time in the disease development and also whether the response is acute or chronic. Activated microglia can reduce Aβ accumulation by increasing its phagocytosis or extracellular degradation [43-45]. Microglia also release trophic factors such as the glia-derived neurotrophic factor (GDNF), which is neuroprotective [46]. In addition, certain cytokines have an anti-inflammatory effect, such as IL-1 receptor antagonist (IL-1Ra), IL-4, IL-10 and TGF-β [22,47]. It was recently reported that the interaction between newly formed amyloid plaque and microglia shows that, unless further activated, microglia do not successfully clear plaques but may well restrict their growth, leading to a steady state of plaque size after initial formation [10].

Further support for the involvement of inflammation with the pathogenesis of AD came from the finding in epidemiological studies that treatment with non-steroidal anti-inflammatory drugs (NSAIDs) was associated with a reduced risk of developing AD. A meta-analysis of nine studies revealed that the benefit was greater with long-term use than with intermediate use [48]. Recently, a modified Mini-Mental State Examination reported that NSAIDs use prevented cognitive decline in older adults if started in midlife (prior to age 65) rather than late in life (after age 65). This effect was more pronounced in those who had one or more APOE e4 alleles [49]. However, the possible preventive effect of NSAIDs has not been yet confirmed in clinical trials. The failure of the trials may be attributed to the facts that the benefit of NSAIDs may only be observed in early phases of the disease, that is, they are preventive and not curative, and also to the choice of NSAID, mostly COX-2-specific inhibitors because (i) only a subset of NSAIDs are able to lower Aβ production and (ii) this capacity appears to be COX-2 independent [50]. In animals, the beneficial effects of NSAIDs have been confirmed, showing behavioural improvement and reductions in glial activation, in Aβ levels and plaque size [45,51-55]. In vitro studies have revealed several potential targets for NSAIDs, including NFκB, Rho-GTPases, PPARγ and secretases [50]. However, the inhibition of the canonical targets of NSAIDs, cyclooxygenase-1 and -2 (COX-1 and COX-2), do not seem to be responsible for the protective effect of NSAIDs in AD. On the contrary, COX-2 inhibitors may raise Aβ1–42 secretion [56].

The finding that NSAIDs could affect APP processing and that certain NSAIDs can inhibit β and γ-secretases with the recent revelation that secretases may be influenced by inflammation indicates that they could be an interesting target for anti-inflammatory drugs [50].

## Secretases and inflammation

### α-Secretase

APP is cleaved by α-secretase in the centre of the Aβ domain. Three related metalloproteases of the ADAM (a disintegrin and metalloprotease) family, ADAM-9, ADAM-10 and ADAM-17, also termed TACE (tumor necrosis factor converting enzyme), appear to exert α-secretase activity [16,57]. A confirmation that ADAM10 is involved in α-secretase activity came from studies in transgenic mice. A moderate neuronal over-expression of ADAM10 in mice transgenic for human APP([V717I]) showed increased secretion of the neurotrophic soluble α-secretase-released N-terminal APP domain (α-APPs), reduced formation of Aβ peptides, and prevention of their deposition in plaques [58]. Functionally, in these mice impaired long-term potentiation and cognitive deficits were alleviated. Expression of mutant catalytically inactive ADAM10 led to an enhancement of the number and size of amyloid plaques in the brains of double-transgenic mice. These results provided the first *in vivo *evidence for a proteinase of the ADAM family as an α-secretase of APP, revealed activation of ADAM10 as a promising therapeutic target, and supported the hypothesis that a decrease in α-secretase activity contributes to the development of AD [58]. Another candidate with α-secretase activity is BACE2, which likewise cleaves within the Aβ domain and abrogates Aβ formation [57] (Figure 1).

ADAM proteinases have emerged as the major protein family that mediates ectodomain shedding, the proteolytic release of extracellular domains from their membrane-bound precursors. Proteolytic cleavage or ectodomain shedding is an additional mechanism whereby cells can regulate the proteins expressed on their cell surface. In many cases, soluble ectodomains are biologically active as mediators of functions ascribed to their transmembrane counterparts [59]. ADAM-like sheddases activate, for instance, growth factors and cytokines, thus regulating signalling pathways that are important in development and pathological processes such as cancer [60]. ADAM17 and 10 appear to play a particularly prominent role in ectodomain shedding of inflammatory proteins at all stages of leukocyte recruitment. Soluble TNF-α is released from its membrane-bound precursor by shedding through ADAM17 [61]. *In vivo *studies have shown that many, but not all, of the inflammatory effects of TNF-α require cleavage and shedding from the cell surface. Besides TNF-α, ADAM17 cleaves ectodomains of other receptors and ligands, such as TNFR2 and L-selectin [62]. In addition, it was recently shown that IL-1R2 can be proteolytically processed in a manner similar to APP [63]. IL-1R2 undergoes ectodomain shedding in an α-secretase manner, resulting in the secretion of the IL-1R2 ectodomain and the generation of a C-terminal fragment.

That α-secretase is involved in inflammation is supported by the observation that ADAM-10 is expressed constitutively by astrocytes in the normal and inflamed human CNS [64]. ADAM10 and ADAM-17 are also enriched in microglia [65]. The functions of ADAMs in microglia are complex and they do not only have pro-inflammatory and neurotoxic properties but also reparative ones [66]. In fact, IL-1α has been reported to increase ADAM-17 and ADAM-10 levels and α-secretase activity in human astrocytic cultures [67]. More recently, it has been shown that the proinflammatory cytokines TNF-α, IFN-γ and IL-1β, as well as TGF-β and LPS, are able to increase ADAM10 activity, leading to a loss in E-cadherin expression, which is another substrate for this secretase [68].

On the other hand, it has been also reported that various NSAIDs (including nimesulide, ibuprofen and indomethacin) stimulate the non-amyloidogenic secretion of sAPPα from neuroblastoma cells [69]. Shedding of sAPPα induced by nimesulide and thalidomide was modulated by inhibitors of PKV and Erk MAPK, indicating that NSAIDs activate the Erk MAPK signalling cascade. However, these results have not been reproduced by other groups [70,71].

### β-Secretase

β-Secretase (BACE1 for β-site APP cleaving enzyme) was cloned and identified as a type I transmembrane aspartyl protease [72]. BACE1 cleaves APP at the N-terminal position of Aβ. BACE1 deficiency precludes Aβ formation in transgenic mice, and does not cause or promote any neurological or phenotypic abnormalities. However, it has been recently demonstrated that BACE1 knockout mice exhibit a number of schizophrenia-like behavioural traits, most likely because BACE1 is involved in the cleavage of neuregulin-1, which has been linked to the pathogenesis of schizophrenia and related psychiatric disorders [73]. Because BACE1 inactivation rescues memory deficits in transgenic mice [74], this strongly supports the importance of BACE1 as a therapeutic target in AD.

BACE1 is primarily expressed in neurons [72,75,76], but it can be also expressed in astrocytes under conditions of chronic stress [77] and in old transgenic mice [76,32]. In addition, in young transgenic mice, neuronal BACE1 was induced in the proximity of activated microglia and astrocytes [32]. These observations lead to the conclusion that BACE1 expression is regulated by inflammatory events. BACE1 has also been found up-regulated in neuronal cultures upon exposure to pro-inflammatory cytokines [70,78], under oxidative stress in NT2 neurons [79], and in the hippocampus of rats after experimental traumatic brain injury [39]. In addition, BACE1 protein levels, activity and the β-secretase product (β-CTF) are increased in brain of sporadic AD patients [80,81] as well as in platelets and CSF from AD and MCI patients [82-84]. These alterations in BACE1 expression and activity indicate a transcriptional and/or translational regulation of BACE1 expression in the brain [85]. Interestingly, consensus binding sites for various transcription factors that are known to be regulated by inflammation (such as NFκB, PPARγ and STAT1) are present in the BACE1 promoter.

## Transcriptional regulation

### NFκB

NFκB sites are present in the promoters of APP [86], presenilin and BACE1 [87]. In neurons exposed to soluble Aβ peptides and in TNFα-activated glial cells the mutation of the BACE1 promoter NFκB site led to significant decreases in promoter activity, indicating an activating role for NFκB in BACE1 expression in Aβ [87]. In addition, some NSAIDs such as flurbiprofen and indomethacin, which target NFκB, have been shown to be effective at decreasing amyloid load *in vitro *and also in APP transgenic mice [54,55,88]. A recent report showed that deletion of TNFα1 death receptor (TNFα1R) in APP23 transgenic mice inhibited Aβ generation reducing BACE1 levels and activity via the NFκB pathway [89].

The effect of NFκB on BACE1 promoter could be direct or through changes in PPARγ, because PPARγ agonists can antagonize the activity of transcription factors such as NFκB [50].

### PPARγ

PPARγ is a transcription factor that is involved in the regulation of the metabolism of glucose and lipids, in cellular differentiation as well as in the control of transcription of a wide range of inflammatory genes. A consensus binding site for PPARγ was found in the BACE1 promoter. PPARγ activation by agonists such as thiazolidinediones (TZD) and certain NSAIDs such as ibuprofen, indomethacin and naproxen results in a decrease of BACE1 transcription, expression and activity [70]. Furthermore, lack of PPARγ led to an increase of BACE1 promoter activity [90], which suggested that PPARγ could be a repressor of BACE1.

PPARγ levels are decreased in AD brain, indicating that inflammatory events may decrease PPARγ transcription. Furthermore, *in vitro *experiments have shown that inflammatory cytokines and oxidative stress decrease PPARγ levels. Therefore, these findings suggest the existence of a down-regulation of PPARγ under inflammatory conditions, which would result in an increase in BACE1 transcription and Aβ generation. This effect could be prevented by modulation of PPARγ activity by NSAIDs, which have been shown to increase the levels of PPARγ in adipocytes and neurons [90,53].

### STAT-1

STAT-1 can bind directly to a putative STAT1 binding sequence in the BACE1 promoter. A recent study showed that when STAT1 becomes phosphorylated by IFNγ-mediated activation of JAK2 and ERK1/2, STAT1 binds to BACE1 promoter region to increase BACE1 protein expression in astrocytes [91]. On the other hand, another report has demonstrated that IFNγ-receptor knockout mice crossed with Tg2576 mice expressing the human Swedish mutant APP had reduced gliosis and amyloid plaque compared with Tg2756 animals, apparently by decreasing TNFα secretion and the number of reactive astrocytes expressing BACE1 in cortex and hippocampus [78].

## Post-transcriptional modifications

Evidence has been presented for regulation of BACE1 expression at the translational level. The untranslated 5'-BACE1 transcript leader contains upstream open reading frames (uORF) that can reduce the translation of the main open reading frame [92,93]. It remains unclear as to whether BACE1 translation is constitutively repressed or whether the repression may be alleviated upon physiological and pathophysiological stimuli [85]. Potentially, the identification of proteins specifically binding to long, GC-rich 5'UTR may yield new insights into the possible regulation of BACE1 translation. Evidence for these mechanisms was shown by *Zacchetti *et al., who described that the 5'-UTR-dependent translational repression of BACE1 may be alleviated in activated astrocytes [94]. Therefore, it seems that inflammation may play also a role in the translational regulation of BACE1.

### γ-Secretase

γ-Secretase is a protein complex of four essential membrane proteins called aph-1, pen-2, nicastrin and presenilin (PS). While aph-1, pen-2 and nicastrin function in the assembly and subcellular transport of γ-secretase and in the recognition of protein substrates [95-97]. PS proteins are members of an aspartyl protease family and represent the catalytically active components of the γ-secretase complex [98]. PS1 and PS2 proteins are homologous polytopic membrane proteins critically involved in intra-membraneous cleavage of APP and a number of other type I membrane proteins [98-100]. Accordingly, PS proteins have been implicated in different biological processes, including the regulation of cell differentiation and death, calcium homeostasis, cell adhesion, and subcellular trafficking of several membrane proteins. Mutations in PS are associated with familial forms of early onset Alzheimer's disease (AD) and increase the ratio of Aβ42/Aβ40 by either elevating production of the elongated, highly fibrillogenic Aβ42 or by decreasing the generation of Aβ40 [57,101,102].

### γ-Secretase and inflammation

It was recently published that activated microglia and astrocytes have enhanced expression of PS and nicastrin following brain damage [42]. Although glial cells express PS1, it is not known if PS1 mutations alter glial cell functions. Carriers of PS FAD associated mutations not only show earlier deposition of Aβ in β-amyloid plaques, but also inflammatory processes in the brain [90]. On the other hand, it is unclear whether PS FAD associated mutations cause neuroinflammation by promoting formation and deposition of Aβ42 or by triggering other processes. In PS1 associated FAD brain, distinct 'inflammatory plaques' have been described, that lack reactivity for apolipoprotein E and Aβ in the core, but revealed association with reactive microglia and astrocytes, suggesting that mutations in PS1 could also induce Aβ independent neuroinflammation in Early Onset Alzheimer's disease (EOAD) [103]. In addition, studies using knock-in mice for PS1 FAD mutations have revealed an enhanced inflammatory cytokine response to immune challenge with bacterial lipopolysaccharide (LPS). LPS-induced levels of mRNAs encoding TNFα, IL-1α, IL-1β, IL-1 receptor antagonist, and IL-6 were significantly greater in the hippocampus and cerebral cortex of PS1 mutant mice as compared to wild-type mice. These findings demonstrate an adverse effect of PS1 mutations on microglial cells that results in their hyperactivation under pro-inflammatory conditions, which may, together with direct effects of mutant PS1 in neurons, contribute to the neurodegenerative process in AD [104].

Additional evidence for an Aβ independent role of PS proteins in inflammation comes from studies with conditional knockout mice lacking both PS genes in the postnatal forebrain. These mice display strong age-dependent neurodegeneration and impairment of cognitive function [105]. Gene profiling revealed up-regulation of several pro-inflammatory genes, including glial fibrillary acidic protein, complement component C1q, and cathepsin S. Also, activated microglia were detected in the brain of these mice. Since Aβ production is strongly reduced upon deletion of PS in forebrain neurons, these data indicate that impairment of PS function could also trigger inflammation independent of Aβ [105]. Analogously, the cleavage of other substrates for PS, such as Notch, could also have some effect on brain inflammation. It was shown that mice transgenic for antisense Notch and normal mice treated with inhibitors of γ-secretase showed reduced damage to brain cells and improved functional outcome in a model of focal ischemic stroke. These mice had a reduced number of activated microglial cells in the brain after ischemic perfusion [106].

### NSAIDs and γ-secretase

As described above several NSAIDs decrease the risk for development of AD. Although the molecular mechanism(s) underlying this protective activity remain to be identified, certain NSAID could directly modulate γ-secretase activity [107]. Thus, besides suppression of inflammatory processes via inhibition of COX dependent biosynthesis of pro-inflammatory prostaglandins, the protective role of NSAIDs could also involve altered generation of Aβ. Indeed, several NSAIDs, including ibuprofen, sulindac sulphide, and indomethacin could decrease the levels of secreted Aβ42 [108,54,109]. Importantly, generation of Aβ40 is largely unaltered by these compounds, indicating that certain NSAIDs modulate rather than inhibit γ-secretase activity. The decrease in Aβ42 production was associated with increased generation of Aβ38 suggesting a shift in the cleavage specificity of the protease [107]. However, more recently it has been shown that the generation of Aβ38 and Aβ42 is independent and differently affected by FAD mutations in PS as well as by modulators of γ-secretase [110]. After the initial identification of ibuprofen, sulindac sulphide, and indomethacin as selective Aβ42 lowering drugs, several other NSAIDs, including flurbiprofen and fenoprofen have been shown to exert similar effects [54]. Because Aβ42 lowering activity was also observed with several derivatives of NSAIDs that do not inhibit COX, and was also present in COX1/2 deficient cell lines, the modulation of γ-secretase activity is unlikely to be mediated via COX enzymes [107].

Surprisingly, the PPARα antagonist fenofibrate and some COX-2 specific inhibitors could even increase Aβ42 production [56,111,112]. Whether additional effects of NSAIDs on NFκB and PPARα or PPARγ (see above) contribute to the Aβ42 lowering activity remains to be investigated in more detail. However, certain NSAIDs decrease Aβ42 production in vitro with purified γ-secretase complex, indicating NFκB and PPARγ independent effects [113,114]. Interestingly, FAD associated mutations in PS1 could cause insensitivity of γ-secretase to Aβ42 lowering NSAIDs [115]. Because direct interaction of NSAIDs with the presenilins or other components of the γ-secretase complex has not been shown so far, the molecular mechanisms for the action of NSAIDs are unclear. Since NSAIDs reveal Aβ42 lowering activity at relatively high doses (IC_50 _values of 25 – 500 μM) at which they could also affect biophysical properties of biological membranes, the modulation of γ-secretase specificity might also involve interference with membrane fluidity and/or accessibility of substrate to the enzyme [116]. Nonetheless, since NSAIDs, even at higher concentrations did not inhibit cleavage of Notch and ErbB4 or alter AICD formation, this class of inhibitors might be valuable compounds to selectively decrease Aβ42 production without affecting important intracellular signaling pathways, thereby reducing potential side effects in clinical applications.

In preclinical studies, long-term treatment of APP transgenic Tg2576 mice with ibuprofen and indomethacin were effective in reducing the plaque pathology [51,117]. Also, acute treatment with additional NSAIDs, sulindac sulfide, and flurbiprofen, selectively reduced Aβ42 levels without changing the concentration of Aβ40 [54,108]. However, other studies did not reproduce these results [111,118]. In clinical trials some positive effects on cognitive performance of AD patients were observed with indomethacin and the (R)-enantiomer of flurbiprofen, while other NSAIDs without Aβ42 reducing activity did not reveal beneficial effects [107] (see Table 1 for summary). Although this indicates that it would be relevant to evaluate Aβ42 lowering NSAIDs in further clinical trails, recent epidemiological studies revealed that the protective effect of NSAIDs seems to be independent of the Aβ42 suppressing activity of the NSAID [119,120].

**Table 1:** Comparison of the effects of NSAIDs on APP secretases. The decrease in BACE1 was detected under inflammatory conditions.

**NSAIDs**	**activity**	**α-secretase**	**β-secretase**	**γ-secretase**
Ibuprofen	PPARγ activator	↑(69)↕ (70,71)	↓ (70,90)	↓Aβ42/Aβ40 (108)
Indomethacin	PPARγ activator NFκB inhibitor	↑ (69) ↕ (71)	↓ (70,90)	↓Aβ42/Aβ40 (108)
Naproxen	PPARγ activator	?	↓ (70,90)	↕ Aβ42/Aβ40 (108)
Flurbiprofen	NFκB inhibitor	?	?	↓Aβ42/Aβ40 (54,108)
Aspirin	COX-1 inhibitor	?	↕ (90)	↕ Aβ42/Aβ40 (108)
Sulindac sulphide	COX-1 inhibitor	?	↕ (90)	↓Aβ42/Aβ40 (108)
Celecoxib	COX-2 inhibitor	?	↕ (70,90)	↑Aβ42/Aβ40 (56)
Fenofibrate	PPARα activator	?	?	↑Aβ42/Aβ40 (56)

## Conclusion

**Figure 2:**
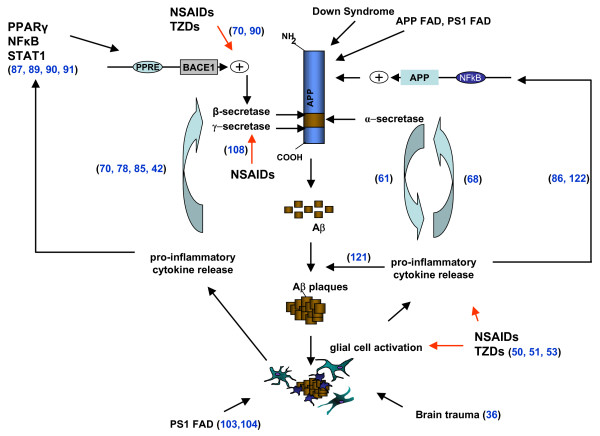
Schematic representation of the interactions between inflammatory processes and APP processing. Aβ generation by BACE1 and γ-secretase induces an inflammatory response, which involves the activation of microglia and astrocytes and the release of pro-inflammatory cytokines. This inflammatory response could be enhanced by brain trauma or by PS1 mutations, probably also by increased Aβ production. Inflammatory cytokines have been involved in the aggregation of Aβ [121]. Moreover, cytokines can affect the expression of secretases and APP, influencing their transcription, translation and/or activation [122]. Non-steroidal anti-inflammatory drug treatment could reverse the effect of inflammation on BACE1 transcription, modulate the cleavage site of γ-secretase and decrease the secretion of cytokines and the number of microglia and astrocytes. On the other hand, α-secretase has been involved in the shedding of certain cytokines, potentiating their activity. See text for further details of individual interactions. Numbers on the diagram correspond to the appropriate references in the review.

In summary, in this review we have tried to give a perspective on the wide variety of interactions between inflammatory mediators and APP secretases. On the one hand, pro-inflammatory cytokines are able to increase the levels and/or activity of some secretases, such as α-secretase and BACE1. On the other hand, NSAIDs are able to modulate the activity of the secretases by regulating their levels in the case of BACE1, or by directly shifting the cleavage site of γ-secretase (see Table 1). In addition it now seems that α-secretase plays a particularly prominent role in ectodomain shedding of inflammatory proteins, thus regulating the activity of cytokines such as TNFα. Bringing all this together, it is clear that the association between inflammation and AD, as suggested by the wealth of epidemiological, clinical and laboratory data, is based on a series of complex molecular interactions that we are only just beginning to understand in detail (Figure 2). The initial signs are encouraging but further work is needed in this area to determine whether modification of these interactions can provide a viable therapeutic target for the treatment of AD.

## List of abbreviations

ADAM: a disintegrin and metalloprotease; ApoE: Apolipoprotein E; COX: cyclooxygenase; EOAD: Early Onset Alzheimer's Disease; FAD: Familiar Alzheimer's Disease; IL: Interleukin; iNOS: inducible nitric oxide synthase; MCI: mild cognitive impairment; NFκB: nuclear factor kappa B; NSAIDs: Non-steroidal anti-inflammatory drugs; PPAR: Peroxisome Proliferator-activated receptor; PS: presenilin; STAT: Signal transducers and activators of transcription; TNFα: Transforming Necrosis Factor-α; TZD: thiazolidinedione

## Competing interests

The authors declare that they have no competing interests.

## Authors' contributions

MS wrote most of the first draft, JW wrote the part of γ-secretase and SMG wrote the section on trauma. All authors were involved in the design of the figures and editing of the manuscript.
